# Feasibility and reference values of left atrial longitudinal strain imaging by two-dimensional speckle tracking

**DOI:** 10.1186/1476-7120-7-6

**Published:** 2009-02-08

**Authors:** Matteo Cameli, Maria Caputo, Sergio Mondillo, Piercarlo Ballo, Elisabetta Palmerini, Matteo Lisi, Enzo Marino, Maurizio Galderisi

**Affiliations:** 1 Department of Cardiovascular Diseases, University of Siena, Siena, Italy; 2 Cardiology Operative Unit, S Andrea Hospital, La Spezia, Italy; 3 GE Healthcare, Milan, Italy; 4 Cardioangiology Unit with CCU, Department of Clinical and Experimental Medicine, Federico II University Hospital, Naples, Italy

## Abstract

**Background:**

The role of speckle tracking in the assessment of left atrial (LA) deformation dynamics is not established. We sought to determine the feasibility and reference ranges of LA longitudinal strain indices measured by speckle tracking in a population of normal subjects.

**Methods:**

In 60 healthy individuals, peak atrial longitudinal strain (PALS) and time to peak longitudinal strain (TPLS) were measured using a 12-segment model for the left atrium. Values were obtained by averaging all segments (global PALS and TPLS) and by separately averaging segments measured in the two apical views (4- and 2-chamber average PALS and TPLS).

**Results:**

Adequate tracking quality was achieved in 97% of segments analyzed. Inter and intra-observer variability coefficients of measurements ranged between 2.9% and 5.4%. Global PALS was 42.2 ± 6.1% (5–95° percentile range 32.2–53.2%), and global TPLS was 368 ± 30 ms (5–95° percentile range 323–430 ms). The 2-chamber average PALS was slightly higher than the 4-chamber average PALS (44.3 ± 6.0% vs 40.1 ± 7.9%, p < 0.0001), whereas no differences in TPLS were found (p = 0.93).

**Conclusion:**

Speckle tracking is a feasible technique for the assessment of longitudinal myocardial LA deformation. Reference ranges of strain indices were reported.

## Background

The left atrium plays as a booster pomp during late ventricular diastole, as a reservoir for the inflow volume received from pulmonary veins during ventricular systole and isovolumic relaxation, and as a passive conduit during early ventricular diastole and diastasis [1,2]. Although estimates of left atrial (LA) function can be obtained by two-dimensional echocardiography, Doppler analysis of transmitral and pulmonary vein flow, and Tissue Doppler (TD) assessment of LA myocardial velocities [3-5], quantification of effective LA function still remains a challenging issue. Assessment of atrial deformation profiles obtained using Doppler-derived strain imaging has been recently proposed as an alternative method of exploring LA function[6]. However, this approach is limited by a number of potential drawbacks, including suboptimal reproducibility, angle dependence, and the confounding effect of noise artifacts[7].

Two-dimensional strain imaging is an echocardiographic technique that uses standard B-mode images for speckle tracking analysis. The speckle pattern (acoustic backscatter generated by the reflected ultrasound beam) is followed frame-by-frame, using a statistical approach based on the detection of the best matching area. The displacement of this speckled pattern is considered to follow myocardial movement, and a change between speckles is assumed to represent myocardial deformation[8,9].

Quantification of LA myocardial function by speckle tracking has been recently proposed[10,11], but data on reference values of LA speckle tracking indices are still lacking.

The aims of this study were to define the feasibility of speckle tracking-based strain imaging for the evaluation of LA wall deformation in a population of healthy subjects, and to identify normality ranges for corresponding strain values.

## Methods

### Study population

Sixty consecutive adult healthy subjects, referring to our Echo Laboratory for a diagnostic examination, were included in the study group. All had unremarkable clinical history and normal findings at physical examination, ECG, and baseline echocardiography, and none of them was taking cardiac medications. All subjects gave their written informed consent for participation in the study.

### Standard echocardiography

Echocardiographic studies were performed using a high-quality echocardiograph (Vivid 7, GE, USA). Subjects were studied in the left lateral recumbent position. Measurements of left ventricular (LV) and LA dimensions were made in accordance with current American Society of Echocardiography recommendations[12]. LV ejection fraction was measured using the modified biplane Simpson's rule. The ratio between peak early (E) and late (A) diastolic LV filling velocities and E wave deceleration time were determined by standard Doppler imaging. The timings of mitral and aortic valve opening and closure were defined by pulsed wave Doppler tracings of mitral inflow and LV outflow.

### Speckle tracking

For speckle tracking analysis, apical four- and two-chamber views images were obtained using conventional two-dimensional gray scale echocardiography, during breath hold with a stable ECG recording. Particular attention was given to obtain an adequate gray scale image, allowing reliable delineation of myocardial tissue and extracardiac structures. Three consecutive heart cycles were recorded and averaged. The frame rate was set between 60 and 80 frames per second. These settings are recommended to combine temporal resolution with adequate spatial definition, and to enhance the feasibility of the frame-to-frame tracking technique[13].

**Figure 1:**
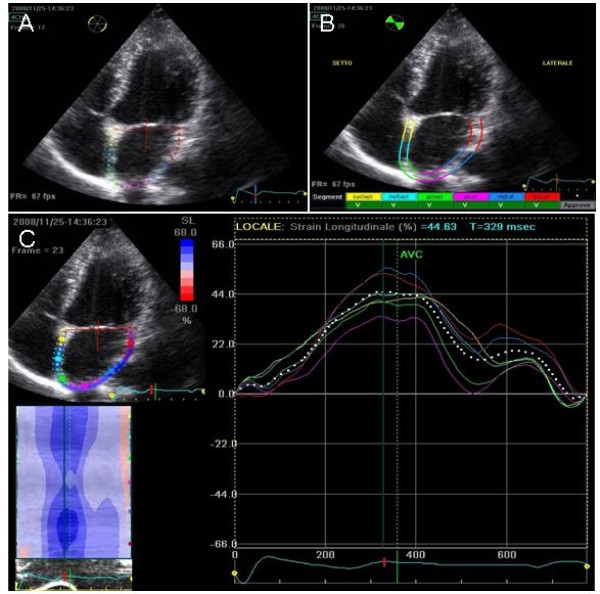
**Measurement of left atrial longitudinal strain by speckle tracking**. A) The atrial endocardial border is traced by a point-and-click method; B) after automatic creation of a region of interest divided in 6 subregions, segmental tracking quality is analyzed; C) after approval by the user, segmental longitudinal strain curves are generated. The dashed curve represents the average strain.

**Figure 2:**
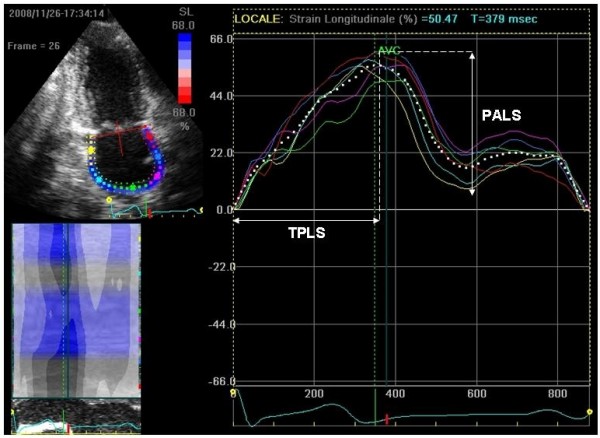
**Measurement of peak atrial longitudinal strain (PALS) and time to peak longitudinal strain (TPLS)**.

Recordings were processed using an acoustic-tracking software (Echo Pac, GE, USA), allowing off-line semi-automated analysis of speckle-based strain[14,15] (Figure 1). Briefly, LA endocardial surface is manually traced in both four- and two-chamber views by a point-and-click approach. An epicardial surface tracing is then automatically generated by the system, thus creating a region of interest (ROI). After manual adjustment of ROI width and shape, the software divides the ROI into 6 segments, and the resulting tracking quality for each segment is automatically scored as either acceptable or non-acceptable, with the possibility of further manual correction. Segments in which no adequate image quality can be obtained are rejected by the software and excluded from the analysis. Lastly, the software generates strain curves for each atrial segment. In subjects with adequate image quality, a total of 12 segments were then analyzed. To trace the ROI in the discontinuity of LA wall corresponding to pulmonary veins and LA appendage, the direction of LA endocardial and epicardial surfaces at the junction with these structures was extrapolated. Peak atrial longitudinal strain (PALS) was calculated by averaging values observed in all LA segments (global PALS), and by separately averaging values observed in 4- and 2-chamber views (4- and 2-chamber average PALS) (Figure 2). The time to peak longitudinal strain (TPLS) was also measured as the average of all 12 segments (global TPLS) and by separately averaging values observed in the two apical views (4- and 2-chamber average TPLS). In patients in whom some segments were excluded because of the impossibility of achieving adequate tracking, PALS and TPLS were calculated by averaging values measured in the remaining segments.

Reproducibility of PALS and TPLS measurements was assessed in 20 randomly selected subjects. Intra and inter-observer variability coefficients were calculated using images independently recorded in two different occasions by the same investigator or by two different observers.

### Statistical analysis

Data are shown as mean ± SD. Inter- and intra-observer reproducibility was assessed by calculating variability coefficients. Reference values were expressed as mean ± SD and 5–95° percentile ranges. Comparisons were performed using the Student's t test for paired data. A P value < 0.05 was considered statistically significant. Analyses were performed using the SPSS (Statistical Package for the Social Sciences, Chicago, Illinois) software Release 11.5.

## Results

### Feasibility

Table 1 shows the clinical and echocardiographic characteristics of the study population. Among a total of 720 segments, the software was able to track 697 (96.9%) segments. Adequate tracking of all 12 LV segments was achieved in 50 (83.3%) subjects, and in no cases the number of segments adequately explored was < 8. Average post-processing time per patient was 2 ± 1 min. Inter-observer variability coefficients of global PALS and TPLS were 3.4%, and 4.8%, respectively. For intra-observer variability, the corresponding variability coefficients were 2.9% and 3.8%.

**Table 1:** General characteristics of the study population

**Variable**	
**Age **(years)	32.8 ± 13.6
**Female gender **(n)	29 (48.3%)
**Height **(cm)	171.3 ± 8.1
**Weight **(kg)	66.7 ± 9.8
**Body surface area **(m^2^)	1.73 ± 0.4
**Body mass index **(kg/m^2^)	22.6 ± 2.2
**Systolic blood pressure **(mmHg)	120 ± 10.7
**Diastolic blood pressure **(mmHg)	77.3 ± 5.2
**Heart rate **(bpm)	73.3 ± 10.0
**End-diastolic LV diameter **(mm)	44.9 ± 4.8
**End-systolic LV diameter **(mm)	28.3 ± 4.2
**LV ejection fraction **(%)	60.0 ± 4.3
**Left atrial diameter **(mm)	28.6 ± 4.8
**Left atrial area **(cm^2^)	13.5 ± 2.5
**Mitral E/A ratio**	1.54 ± 0.4
**Deceleration time **(ms)	283.3 ± 61.0

Clinical and echocardiographic features of the study population. LV = left ventricular.

When the reproducibility was separately considered in the two apical views, inter-observer variability coefficients were 4.3% and 4.6% for 4- and 2-chamber average PALS, and 5.4% and 5.3% for TPLS, respectively. Corresponding intra-observer variability coefficients were 3.6%, 4.0%, 4.5%, and 4.8%.

### Reference ranges

Mean ± SD and 5–95° percentile ranges of global, 4-chamber, and 2-chamber PALS and TPLS observed in the study population are reported in Table 2. Notably, 2-chamber average PALS was significantly higher than 4-chamber average PALS (p < 0.0001), whereas there was no difference between 2- and 4-chamber average TPLS (p = 0.93).

**Table 2:** Reference ranges of left atrial strain indices

**Variable**	**Mean ± SD**	**5–95° Percentile range**
**PALS **(%)		
**Global**	42.2 ± 6.1	32.2–53.2
**Four-chamber average**	40.1 ± 7.9	29.0–53.6
**Two-chamber average**	44.3 ± 6.0	35.2–52.7
**TPLS **(ms)		
**Global**	368.0 ± 29.9	322.9–430.4
**Four-chamber average**	364.2 ± 42.6	300.8–436.9
**Two-chamber average**	367.4 ± 34.1	326.4–435.2

Reference values of global, 4-chamber, and 2-chamber peak atrial longitudinal strain (PALS) and time to peak strain (TPLS) in the study population.

## Discussion

In this study, speckle tracking imaging was found to be a feasible and reproducible method to assess LA longitudinal strain in healthy subjects. The reproducibility of measurements was good, with lower variability in comparison with that obtained by Doppler-derived LA strain imaging[16]. These data suggest that speckle tracking may be considered a promising tool to explore LA myocardial deformation dynamics. Data observed in our population were also used to provide reference ranges for speckle-based LA strain indices.

**Figure 3:**
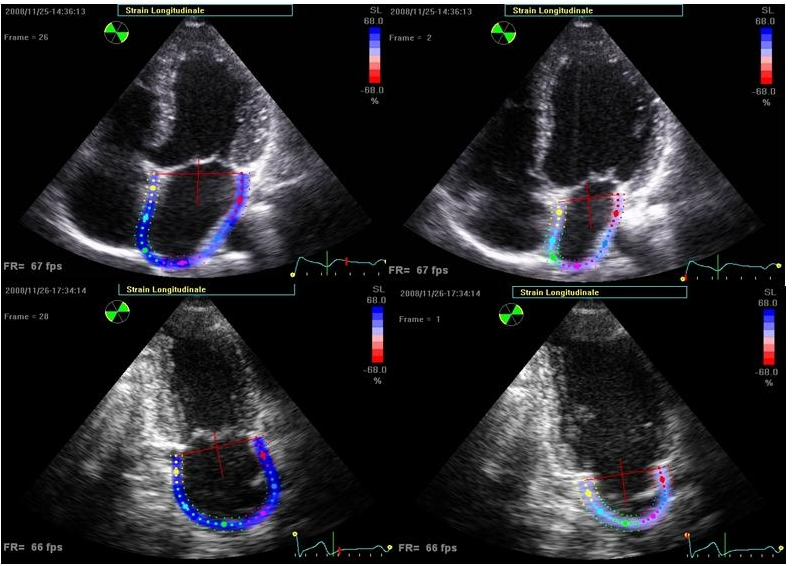
**End-systolic (left panels) and end-diastolic (right panels) frames showing colour-coded left atrial longitudinal strain in a representative subject from both apical views**.

The strain curve evaluated by either Doppler method or speckle tracking imaging closely follows LA physiology (Figure 3, Additional file 1). During the period of LA reservoir (corresponding to the phases of LV isovolumic contraction, ejection, and isovolumic relaxation), LA strain increases, achieving a peak at the end of LA filling from the venous district, just before mitral valve opening. During the conduit phase, LA strain decreases, showing a plateau during diastasis, and achieving a negative peak at the end of LA contraction. Considering the limitations of classical indices of LA function[17], assessment of LA strain by speckle tracking may represent a relatively rapid and easy-to-perform technique to explore LA function. This approach may be of potential clinical importance in a number of pathophysiologic conditions typically associated to abnormal LA function, e.g. mitral valve diseases, supraventricular arrhythmias, systemic hypertension, ischemic heart disease, heart failure, atrial stunning, and cardiomyopathies.

In our population, average peak longitudinal strain was higher in apical 2-chamber view than in apical 4-chamber view. It can be hypothesized that the confounding effect of atrial septum and pulmonary veins ostia in the 4-chamber view – two zones where strain profile is abnormal – may have played a role in determining this discrepancy. In contrast, the average time to peak longitudinal strain was similar in the two apical views. Interestingly, these findings are in contrast with a recent report on LA strain measured by the Doppler-derived technique, which found similar peak strain values among different LA walls, but different timing measures[16]. These discrepancies should be evaluated in further studies. It should be noted that in contrast to Doppler-derived strain imaging, speckle tracking has the advantage of being angle-independent, and to be less affected by reverberations, side lobes and drop out artifacts[8]. Nonetheless, intrinsic limitations of speckle tracking include strict frame rate dependency, potential errors in epicardial/endocardial border tracing in subjects with suboptimal image quality, and need for an appropriate learning curve to achieve adequate experience in using analysis softwares.

Some limitations should be considered. The potential difficulty of accurately obtaining a region of interest close enough to the effective shape of the left atrium, and the risk of contamination by signal components arising from structures surrounding the left atrium should also be considered. Although the post-processing time in this study was relatively short, it closely depends on the sonographer's experience. Lastly, because a dedicated software for LA strain analysis has not yet been released, we used the current software for LV analysis to study the LA pattern strain. Future evolutions in this regard may be useful to improve tracking quality of LA myocardial deformation, and to provide a better instrument for the study of LA function. Lastly, it should be emphasized that as for other echocardiographic new technologies, speckle tracking is progressively entering the clinical practice despite no definite data regarding reference ranges and no clear demonstration of clear additive value in particular clinical conditions. The results of this study may contribute to partially fill this gap giving insight on the potential application of speckle tracking to the study of LA function, but further larger analyses are needed.

## Conclusion

In summary, speckle tracking can be considered a feasible and reproducible technique for the assessment of LA longitudinal deformation dynamics. Normal values of longitudinal strain indices were reported.

## Competing interests

The authors declare that they have no competing interests.

## Authors' contributions

MC, MC, ML, and EP were responsible for the collection of data and drafted the manuscript. PB performed the statistical analysis and revised the manuscript for important intellectual content. SM was responsible for the design of the study and revised the manuscript for important intellectual content. EM provided precious support for the acquisition of data in applying the speckle tracking software to the study of left atrial myocardium. MG also revised the manuscript for important intellectual content. All authors read and approved the final manuscript.

## Supplementary Material

Additional file 1 **Movie 1**. Colour-coded left atrial strain assessed by speckle tracking during a representative cardiac cycle. Click here for file
